# *Batwanema* gen. n. and *Chokwenema* gen. n. (Oxyurida, Hystrignathidae), new nematode genera as parasites of Passalidae (Coleoptera) from the Democratic Republic of Congo

**DOI:** 10.3897/zookeys.361.6351

**Published:** 2013-12-11

**Authors:** Jans Morffe, Nayla García

**Affiliations:** 1 Instituto de Ecología y Sistemática, Carretera Varona 11835 e/ Oriente y Lindero, La Habana 19, CP 11900, Calabazar, Boyeros, La Habana, Cuba

**Keywords:** Nematoda, Hystrignathidae, *Batwanema*, *Chokwenema*, Passalidae, Democratic Republic of Congo

## Abstract

Two new genera and species parasitizing passalid beetles from the Democratic Republic of Congo are described. *Batwanema congo* **gen. n. et sp. n.** is characterized by having females with the cervical cuticle armed with scale-like projections, arranged initially in rows of eight elements that gradually divide and form pointed spines toward the end of the spiny region, two cephalic annuli, clavate procorpus and genital tract monodelphic-prodelphic. Two Malagasian species of *Artigasia* Christie, 1934 were placed in this genus as *B. latum* (Van Waerebeke, 1973) **comb. n.** and *B. annulatum* (Van Waerebeke, 1973) **comb. n.** *Chokwenema lepidophorum* **gen. n. et sp. n.** is characterized by having females with the cervical cuticle armed with scale-like projections, arranged initially in rows of eight elements (similar to *Batwanema*) that divide gradually, forming spines; a single cephalic annule cone-like, truncated, moderately inflated; procorpus sub-cylindrical and genital tract didelphic-amphidelphic.

## Introduction

The nematode family Hystrignathidae Travassos, 1920 is well known as a parasite group restricted to the gut of passalid beetles. At present, 29 genera have been described with more than 100 species.

One of these genera: *Artigasia* Christie, 1934 was diagnosed on the basis of presenting spines on the cervical cuticle, a clavate procorpus and a genital system monodelphic-prodelphic (Christie 1934, Adamson and Van Waerebeke 1992). Considering this diagnosis, many species have been described and placed in *Artigasia*. However, current studies on the genus showed that several species differ notably in features previously ignored, but with a great taxonomic value, such as the form of the cephalic end and the arrangement and shape of the cervical spines. Such combination of features appears to support the statement of *Artigasia* as a complex of genera morphologically distinct.

The African fauna of Hystrignathidae is poorly known with a few species belonging to the genera *Artigasia* (with the larger number of species), *Hystrignathus* Leidy, 1850; *Passalidophila* Van Waerebeke, 1973 and *Xyo* Cobb, 1898 (Théodoridès 1955, 1958; Baker 1967, Van Waerebeke 1973, Van Waerebeke and Remillet 1982). Recently, the study of the group in the area was retaken by Morffe and García (2013) with the description of two new genera from the Democratic Republic of Congo: *Kongonema* Morffe & García, 2013 and *Lubanema* Morffe & García, 2013.

As a continuation of the studies on Congolese hystrignathids, the present paper deals with two new genera. One of these is created in order to separate two peculiar species of *Artigasia*.

## Materials and methods

Several specimens of passalid beetles from the Democratic Republic of Congo were examined during a research visit to the Royal Museum of Central Africa, Tervuren, Belgium. Six specimens of *Pentalobus barbatus* (Fabricius, 1801), three of *Didimoides* cf. *parastictus* (Imhoff, 1843) (all the latter from Mongwalu, Ituri province, Democratic Republic of Congo) and four specimens of *Pentalobus* sp. from Bambesa, Uele region, Democratic Republic of Congo were revised for parasitological studies. All of these passalids were collected during the Belgian expeditions to the Congo in the 1930’s and stored in 70% ethanol.

The hosts were dissected by making incisions in both pleural membranes and the last abdominal sternites. Intestines were extracted and kept in Petri dishes with 70% ethanol. The guts were excised and the parasites removed. Nematodes were transferred to anhydrous glycerine via the slow evaporation method and mounted in the same medium. The edges of the coverslips were sealed using nail polish. Measurements were made with a calibrated eyepiece micrometer attached to a compound microscope. De Man’s ratios a, b, c and V% were calculated. Each variable is shown as the range followed by the mean plus standard deviation in parentheses; the number of measurements is also given. Micrographs were taken with an AxioCam digital camera attached to a Carl Zeiss AxioScop 2 Plus compound microscope. Line drawings were made with the softwares CorelDRAW X3 and Adobe Photoshop CS2 using the micrographs as masters. Scale bars of all plates are given in millimeters.

Some specimens were processed for SEM as follows: they were dehydrated in a graded ethanol series, critical point-dried, mounted in aluminum stubs and coated in gold. SEM micrographs were taken at an acceleration voltage of 22–25 kV.

The type material and vouchers are deposited in the Colección Helmintológica de las Colecciones Zoológicas (CZACC), Instituto de Ecología y Sistemática, Havana, Cuba; the Collection of the Royal Museum of Central Africa (RMCA), Tervuren, Belgium; the Royal Belgian Institute of Natural Sciences (RIT), Brussels, Belgium and the Coleçao Helmintologica do Instituto Oswaldo Cruz (CHIOC), Rio de Janeiro, Brazil.

## Systematics

### Family Hystrignathidae Travassos, 1920

#### Genus Batwanema gen. n.

http://zoobank.org/9B039791-CD4A-4932-A5F8-9A5F7A333FE3

http://species-id.net/wiki/Batwanema

##### Generic diagnosis.

Female. Cervical cuticle armed with wide, scale-like projections, arranged initially in rows of eight elements. Scales divide gradually, forming pointed spines toward the end of the spiny region. Lateral alae present. Head bearing eight rounded, paired papillae. Two short, not prominent cephalic annuli next to head, the second slightly longer and wider than the first. Oesophagus with procorpus clavate, its base set-off from the isthmus. Excretory pore post-bulbar. Genital tract monodelphic-prodelphic. Eggs ovoid. Tail subulate.

##### Type species.

*Batwanema congo* Morffe & García, gen. n. et sp. n.

##### Other species.

*Batwanema latum* (Van Waerebeke, 1973) comb. n.; *Artigasia lata* Van Waerebeke, 1973: pag. 13, fig. 90–101.; *Batwanema annulatum* (Van Waerebeke, 1973) comb. n.; *Artigasia annulata* Van Waerebeke, 1973: pag. 13, fig. 84–89.

##### Distribution.

Democratic Republic of Congo, Madagascar.

##### Etymology.

The generic epithet (neuter in gender), is a combination of Batwa, after the pygmy ethnic group that inhabits the D. R. of Congo, and the suffix–nema.

#### Batwanema congo sp. n.

http://zoobank.org/303612B9-C74C-4296-9BC9-8C291145AFD1

http://species-id.net/wiki/Batwanema_congo

Figure 1A–G , 2A–E

##### Type material.

♀ holotype, Democratic Republic of Congo, Ituri province, Mongwalu; in *Pentalobus barbatus*; 5.VI.1939; Lepersonne coll.; CZACC 11.4700. Paratypes: 4 ♀♀, same data as holotype, CZACC 11.4701-11.4704; 3 ♀♀, same data as holotype, RMCA; 1 ♀♀, same data as holotype, RIT820; 1 ♀♀, same data as holotype, CHIOC.

##### Additional material.

Vouchers: 3♀♀,Democratic Republic of Congo, Uele region, Bambesa, 3°28'N, 25°43'E; in *Pentalobus* sp.; 15.V.1937; J. Vrijdagh coll.; CZACC 11.4705-11.4707. 2♀♀, same data as the latter, RMCA.

##### Measurements.

Holotype (female) a = 14.81, b = 6.24, c = 7.90, V% = 50.63, total length = 2.370, maximum body width = 0.160, stoma length = 0.045, procorpus length = 0.290, isthmus length = 0.033, diameter of basal bulb = 0.070, total length of oesophagus = 0.380, nerve ring to anterior end = 0.200, excretory pore to anterior end = 0.550, anus to posterior end = 0.300, eggs = 0.100–0.110×0.045–0.050 (0.106 ± 0.005×0.048 ± 0.003 n = 3).

Paratypes (females) (n = 9) a = 13.20–17.50 (14.72 ± 1.50 n = 9), b = 4.92–5.56 (5.33 ± 0.24 n = 9), c = 6.28–7.48 (6.87 ± 0.40 n = 9), V% = 51.83–54.29 (52.76 ± 0.92 n = 7), total length = 1.820–2.170 (2.013 ± 0.138 n = 9), maximum body width = 0.120–0.160 (0.138 ± 0.015 n = 9), stoma length = 0.040–0.050 (0.045 ± 0.003 n = 9), procorpus length = 0.270–0.320 (0.283 ± 0.016 n = 9), isthmus length = 0.025–0.035 (0.031 ± 0.004 n = 9), diameter of basal bulb = 0.060–0.075 (0.067 ± 0.004 n = 9), total length of oesophagus = 0.350–0.420 (0.378 ± 0.020 n = 9), nerve ring to anterior end = 0.170–0.210 (0.191 ± 0.013 n = 9), excretory pore to anterior end = 0.490–0.550 (0.515 ± 0.026 n = 4), anus to posterior end = 0.260–0.310 (0.293 ± 0.015 n = 9), eggs = 0.098–0.123×0.030–0.053 (0.108 ± 0.006×0.041 ± 0.006 n = 16).

##### Specimens from Bambesa.

Females (n = 5) a = 15.91–21.00 (18.40 ± 2.22 n = 5), b = 5.11–6.10 (5.60 ± 0.40 n = 5), c = 5.39–6.13 (5.68 ± 0.30 n = 5), V% = 49.74–54.30 (51.84 ± 1.65 n = 5), total length = 1.510–1.890 (1.716 ± 0.162 n = 5), maximum body width = 0.080–0.110 (0.094 ± 0.011 n = 5), stoma length = 0.040–0.050 (0.043 ± 0.004 n = 5), procorpus length = 0.185–0.270 (0.227 ± 0.030 n = 5), isthmus length = 0.025–0.038 (0.032 ± 0.005 n = 5), diameter of basal bulb = 0.033–0.058 (0.049 ± 0.010 n = 5), total length of oesophagus = 0.258–0.360 (0.308 ± 0.036 n = 5), nerve ring to anterior end = 0.135–0.180 (0.155 ± 0.019 n = 4), excretory pore to anterior end = 0.380–0.430 (0.397 ± 0.029 n = 3), anus to posterior end = 0.280–0.340 (0.302 ± 0.023 n = 5), eggs = 0.095–0.118×0.033–0.045 (0.110 ± 0.008×0.039 ± 0.005 n = 10).

##### Description.

Body comparatively slender, widening from the base of the second cephalic annule, maximum body diameter at level of the vulva, tapering towards anus. Cuticle markedly annulated in the spiny region, annuli (*ca.* 2 µm) less marked in the rest of body. Cervical region armed with rows of cuticular projections from the end of the second cephalic annule to the base of isthmus. First row consisting of eight wide, rectangular, scale-like cuticular projections. At level of row 3-4, a shallow cleavage at midpoint of the scales becoming deeper and wider towards the posterior region of body, until reaching row 8–9, where each scale is divided in two or three spines, their tips rounded. Next to it, spines become pointed gradually and increase their number: *ca.* 22 elements in the median rows and *ca.* 34 in the last rows. Lateral alae commencing at level of the isthmus, within the spiny region and extending to about three body-widths posterior to the vulva. Sub-cuticular longitudinal striae present. Head set-off from body by a deep groove, bearing eight rounded, less prominent, paired papillae. Amphids lateral, pore-like. Next to head, two short, not prominent cephalic annule; the second slightly wider and longer than the first. Cephalic annuli poorly differentiated one from the other, only by a shallow groove. Mouth circular. Stoma about four head-lengths long, surrounded by an oesophageal collar. Lumen of anterior region of stoma triangular, with a ridge in each side. Oesophagus consisting of a muscular clavate procorpus, well set-off from the isthmus. Basal bulb rounded, valve plate well developed. Intestine simple, sub-rectilinear. Rectum short, anus not prominent. Nerve ring encircling procorpus at about its midpoint. Excretory pore situated at about half of body width posterior to basal bulb. Vulva a median transverse slit near midbody, lips slightly prominent. Vagina muscular, forwardly directed. Genital tract monodelphic-prodelphic. Ovary reflexed behind the excretory pore, distal flexure *ca.* 1.5 body-widths long. Eggs ovoid, shell with eight rough, longitudinal, hardly prominent ridges. Tail conical, subulate, ending in a sharp point. Male unknown.

**Figure 1.**
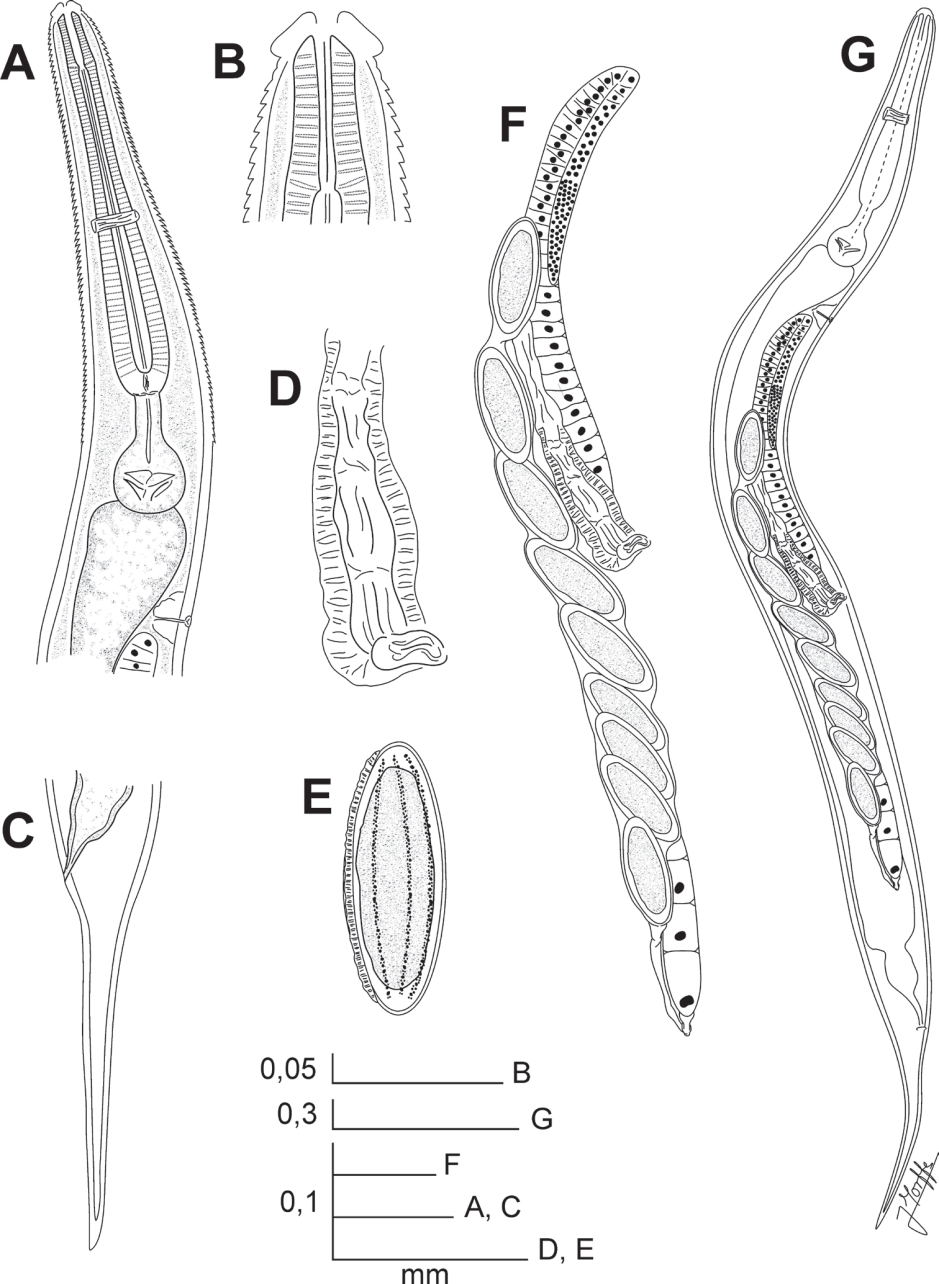
*Batwanema congo* gen. n. et sp. n. Female. **A** Oesophageal region, lateral view **B** Cephalic end, internal view **C** Tail, lateral view **D** Vulva, ventro-lateral view **E** Egg **F** Genital tract, ventro-lateral view **G** Habitus, ventro-lateral view.

**Figure 2.**
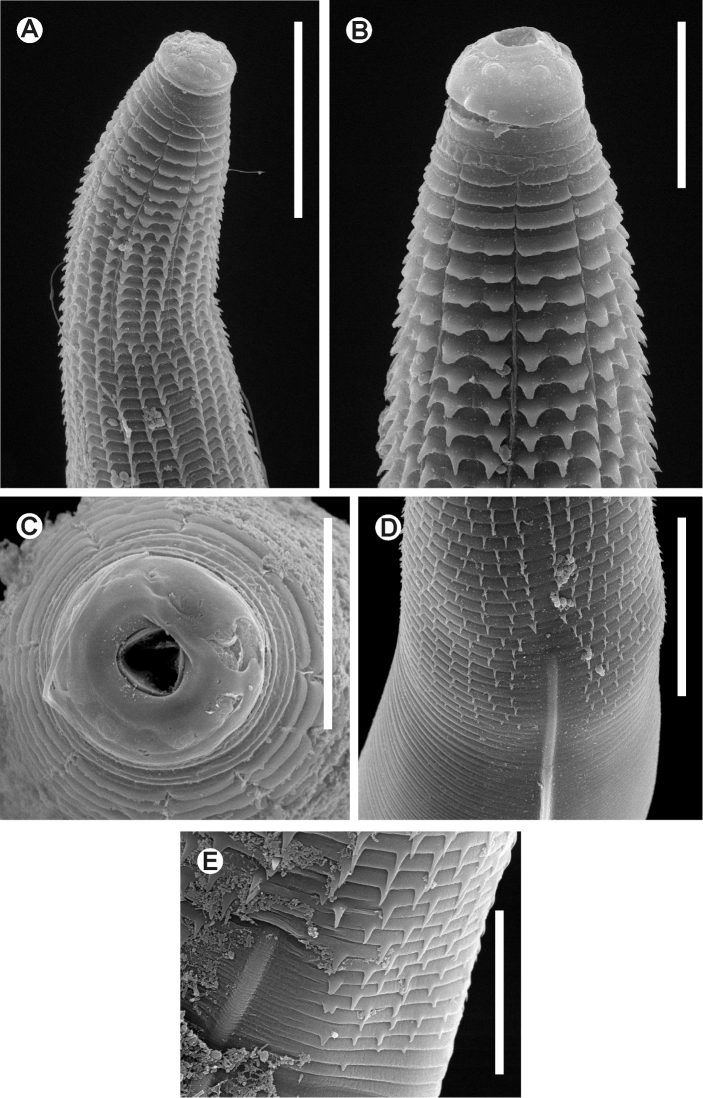
*Batwanema congo* gen. n. et sp. n. Female. SEM images **A** Cervical region **B** Cephalic end, lateralview **C** Cephalic end, *en face* view **D** End of the spiny region and beginning of a lateral ala **E** Detail of the beginning of a lateral ala. Scale lines: **A, D** 0.05 mm, **B** 0.025mm, **C, E** 0.02 mm.

##### Discussion.

*Batwanema* gen. n. presents a similar arrangement of the cervical cuticular projections to *Chokwenema* gen. n., consisting of a first row of eight rectangular scales that gradually bifurcate, becoming pointed spines. It can be differentiated by its reproductive system monodelphic-prodelphic contrary to didelphic-amphidelphic. The genus has two cephalic annuli barely expanded *vs.* the unique truncate, more expanded first cephalic annule of *Chokwenema* gen. n. In addition, the procorpus of *Batwanema* gen. n. is clavate *vs.* sub-cylindrical.

The other genera with scales in the cervical cuticle are *Lepidonema* Cobb, 1898 and *Salesia* Travassos & Kloss, 1958, both having genital tracts didelphic-amphidelphic and with more elements in the first row of spines: 16 *vs.* 8 and a single, large cephalic annuli *vs.* the two shorter of *Batwanema* gen. n. Also, *Lepidonema* has a sub-cylindrical procorpus *vs.* the clavate of *Batwanema* gen. n.

Van Waerebeke (1973) described 14 species of Malagasian *Artigasia*, all of these quite variable in the shape of the cephalic end and the form and arrangement of spines. Two of such species: *Artigasia lata* and *Artigasia annulata* are characterized, in addition to the clavate procorpus and the monogonant genital tract, by the presence of two cephalic annuli (the second larger) and the cervical region with scale-like cuticular projections. These scales are arranged initially in a row of eight elements, which increase their number and become gradually in pointed spines. The latter features agree with the diagnosis of *Batwanema* gen. n., supporting the establishment of *Batwanema latum* comb. n. and *Batwanema annulatum* comb. n. as new combinations of *Artigasia lata* and *Artigasia annulata*.

*Batwanema congo* gen. n. et sp. n. can be segregated from *Batwanema latum* comb. n. by the extension of the cervical spines and lateral alae. In the new species, the spines end at level of the basal bulb *vs.* the level of the nerve ring in *Batwanema latum* comb. n. On the other hand, the lateral alae of *Batwanema congo* gen. n. et sp. n. arise within the spiny region and extend to a distance beyond the vulva, whereas *Batwanema latum* comb. n. presents lateral alae from the beginning of the isthmus to the level of anus. *Batwanema congo* gen. n. et sp. n. has a larger body (1.820–2.370 *vs.* 1.360–1.472) but the oesophagus is comparatively shorter (b = 4.92–6.24 *vs.* 4.10–4.60).

The eggs of both taxa are similar in size (*Batwanema congo* gen. n. et sp. n. = 0.098–0.123×0.030–0.053; *Batwanema latum* = 0.112–0.116×0.039–0.042), but are ridged-shelled in *Batwanema congo* gen. n. et sp. n. *vs.* the smooth-shelled eggs of *Batwanema latum* comb. n. The tail of *Batwanema congo* gen. n. et sp. n. is comparatively longer (c = 6.28–7.90 *vs.* 11.00–13.00).

*Batwanema congo* gen. n. et sp. n. differs from *Batwanema annulatum* comb. n. by the cervical spines extending further down the body, whereas *Batwanema annulatum* comb. n. has spines ending before the level of the basal bulb. Lateral alae of *Batwanema annulatum* comb. n. extend from the level of the isthmus to the level of the anus in opposition to *Batwanema congo* gen. n. et sp. n. where the lateral alae clearly finish before the anus. *Batwanema congo* gen. n. et sp. n. is also longer (1.820–2.370 *vs.* 1.439–1.509), with the oesophagus comparatively shorter (b = 4.92–6.24 *vs.* 4.50–4.60). Moreover, the eggs of *Batwanema annulatum* comb. n. are smooth-shelled *vs.* the ridged-shelled ones of *Batwanema congo* gen. n. et sp. n.

##### Type host.

*Pentalobus barbatus* (Fabricius, 1801) (Coleoptera: Passalidae).

##### Other host.

*Pentalobus* sp. (Coleoptera: Passalidae).

##### Site.

Gut caeca.

##### Type locality.

Mongwalu, Ituri province, Democratic Republic of Congo.

##### Other locality.

Bambesa, Uele region, Democratic Republic of Congo.

##### Etymology.

Specific epithet in apposition refers to the country of the new taxon.

#### Genus Chokwenema gen. n.

http://zoobank.org/CF402D0C-D9CB-47F4-9B52-08BAC87A6D11

http://species-id.net/wiki/Chokwenema

##### Generic diagnosis.

Female. Cervical cuticle armed with wide, scale-like projections, arranged initially in rows of eight elements. Scales divide gradually, forming spines. Head bearing eight rounded, paired papillae. First cephalic annule cone-like, truncated, comparatively long, moderately inflated. Oesophagus with procorpus sub-cylindrical, its base set-off from the short isthmus. Excretory pore post-bulbar. Genital tract didelphic-amphidelphic. Eggs ovoid. Tail subulate.

##### Type species.

*Chokwenema lepidophorum* Morffe & García gen. n. et sp. n.

##### Distribution.

Democratic Republic of Congo.

##### Etymology.

The generic name (neuter in gender) is derived of Chokwe, after an ethnic group from Central Africa (including the D. R. of Congo) and the suffix–nema.

#### Chokwenema lepidophorum sp. n.

http://zoobank.org/124AD063-4948-4F9C-8BD2-F796C14DAA59

http://species-id.net/wiki/Chokwenema_lepidophorum

Figure 3A–H , 4A–C

##### Type material.

♀ holotype, Democratic Republic of Congo, Ituri province, Mongwalu; in *Didimoides* cf. *parastictus*.; 5.VI.1939; Lepersonne coll.; CZACC 11.4708. Paratypes: 3 ♀♀, same data as holotype, CZACC 11.4709-11.4711; 4 ♀♀, same data as holotype, RMCA; 1 ♀♀, same data as holotype, CHIOC.

##### Additional material.

Vouchers: 3♀♀, Democratic Republic of Congo, Ituri province, Mongwalu; in *Pentalobus barbatus*; 5.VI.1939; Lepersonne coll.; CZACC 11.4712-11.4714; 2♀♀, same data as the latter, RMCA; ♀, same data as the latter, RIT821.

##### Measurements.

Holotype (female) a = 11.65, b = 5.25, c = 13.40, V% = 55.97, total length = 2.680, maximum body width = 0.230, first cephalic annule (length×width) = 0.018×0.063, stoma length = 0.038, procorpus length = 0.400, isthmus length = 0.025, diameter of basal bulb = 0.090, total length of oesophagus = 0.510, nerve ring to anterior end = 0.260, excretory pore to anterior end = 0.720, anus to posterior end = 0.200, eggs = 0.093–0.098×0.038–0.040 (0.095 ± 0.003×0.039 ± 0.001 n = 3).

Paratypes (females) (n = 8) a = 9.09–15.63 (10.87 ± 2.40 n = 6), b = 4.26–4.90 (4.62 ± 0.28 n = 5), c = 11.68–12.53 (12.04 ± 0.36 n = 4), V% = 54.40–57.75 (55.76 ± 1.24 n = 6), total length = 2.000–2.500 (2.192 ± 0.181 n = 6), maximum body width = 0.160–0.225 (0.209 ± 0.021 n = 8), first cephalic annule (length×width) = 0.015–0.020×0.058–0.063 (0.018 ± 0.002×0.060 ± 0.002 n = 6), stoma length = 0.030–0.040 (0.035 ± 0.004 n = 7), procorpus length = 0.330–0.420 (0.376 ± 0.032 n = 7), isthmus length = 0.023–0.038 (0.027 ± 0.006 n = 5), diameter of basal bulb = 0.088–0.100 (0.093 ± 0.005 n = 8), total length of oesophagus = 0.450–0.520 (0.487 ± 0.026 n = 6), nerve ring to anterior end = 0.210–0.260 (0.235 ± 0.021 n = 4), excretory pore to anterior end = 0.660, anus to posterior end = 0.170–0.210 (0.184 ± 0.019 n = 4), eggs = 0.090–0.100×0.038–0.050 (0.097 ± 0.004×0.043 ± 0.004 n = 11).

##### Specimens from

***Pentalobus barbatus*.** Females (n = 6) a = 10.36–15.00 (13.25 ± 1.85 n = 6), b = 4.94–5.80 (5.41 ± 0.31 n = 6), c = 13.35–15.73 (14.21 ± 0.95 n = 6), V% = 51.31–56.99 (53.49 ± 2.40 n = 5), total length = 2.175–2.720 (2.453 ± 0.228 n = 6), maximum body width = 0.170–0.210 (0.187 ± 0.016 n = 6), first cephalic annule (length×width) = 0.015–0.018×0.048–0.058 (0.016 ± 0.001×0.053 ± 0.004 n = 6), stoma length = 0.033–0.038 (0.036 ± 0.002 n = 6), procorpus length = 0.310–0.390 (0.353 ± 0.027 n = 6), isthmus length = 0.018–0.033 (0.025 ± 0.006 n = 5), diameter of basal bulb = 0.083–0.103 (0.091 ± 0.007 n = 6), total length of oesophagus = 0.410–0.490 (0.453 ± 0.029 n = 6), nerve ring to anterior end = 0.210–0.250 (0.228 ± 0.015 n = 5), anus to posterior end = 0.150–0.200 (0.173 ± 0.022 n = 6), eggs = 0.088–0.100×0.035–0.050 (0.095 ± 0.004×0.042 ± 0.004 n = 10).

##### Description.

Female body robust, widening from the base of the first cephalic annule, maximum body diameter at level of the vulva, then tapering towards anus. Cuticle markedly annulated in the spiny region, annuli less marked in the rest of the body (*ca.* 3–5µm). Cervical cuticle armed initially by opposite rows of rectangular scales, arranged in number of eight. Scales bifurcate gradually at the third row by a cleavage. Division is total at level of the fifth row, with 16 shorter scales, their tips rounded. Scales becoming pointed towards the end of the spiny region. Last rows of spines (a total of 35–36) end at about one seventh of the body-width before the base of the procorpus. Sub-cuticular longitudinal striae present. Lateral alae absent. Head bearing eight rounded, paired papillae. Amphids lateral, pore-like. First cephalic annule similar in length to head, cone-like, truncated, slightly inflated. Stoma short, about two first cephalic annule-lengths long, surrounded by an oesophageal collar. Oesophagus consisting of a muscular, sub-cylindrical procorpus, its base well set-off from the short isthmus. Basal bulb pyriform, valve-plate well developed. Intestine simple, sub-rectilinear. Rectum short, anus not prominent. Nerve ring encircling the procorpus at *ca.* 55% of its length. Excretory pore located at *ca.* three fourths of the body-width posterior to the basal bulb. Vulva a median transverse slit, displaced to the posterior half of body, its lips slightly prominent. Vagina muscular, directed forwardly. Genital tract didelphic-amphidelphic, both ovaries reflexed. Oocytes in single rows. Eggs comparatively small, ovoid, with eight rough longitudinal ridges in the shell. Tail short, conical, subulate, ending in a sharp tip. Male unknown.

**Figure 3.**
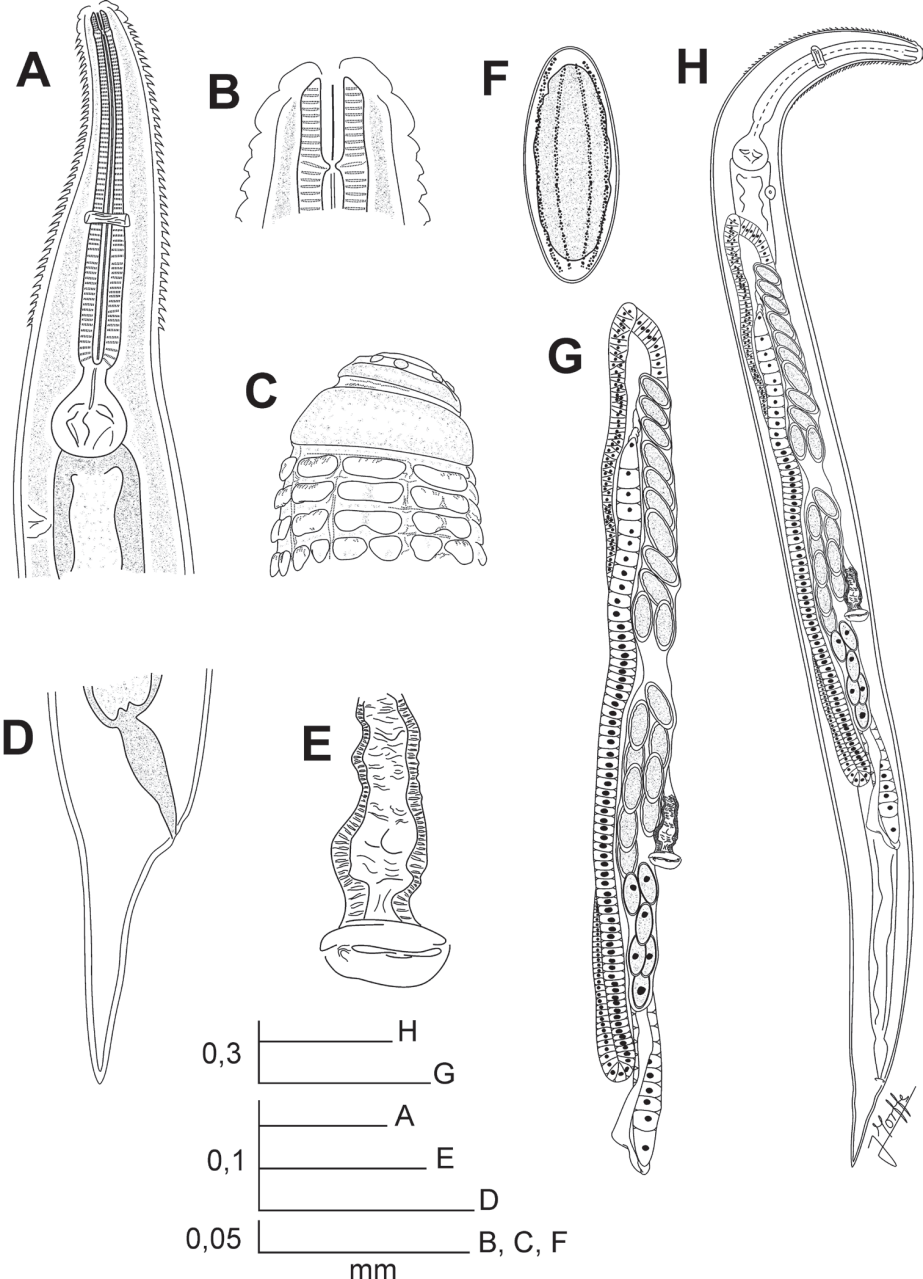
*Chokwenema lepidophorum* gen. n. et sp. n. Female. **A** Oesophageal region, lateral view **B** Cephalic end, internal view **C** Cephalic end, external view **D** Tail, lateral view **E** Vulva, ventral view **F** Egg **G** Genital tract, ventro-lateral view **H** Habitus, ventro-lateral view.

**Figure 4.**
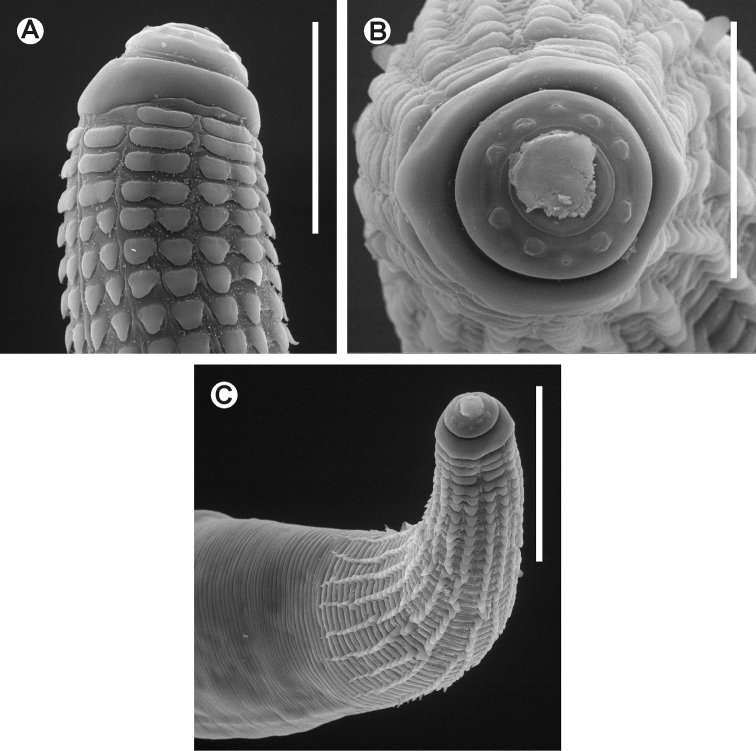
*Chokwenema lepidophorum* gen. n. et sp. n. Female. SEM images **A** Cephalic end **B** Cephalic end, *en face* view **C** Cervical region. Scale lines: **A** 0.05 mm, **B** 0.04mm, **C** 0.1 mm.

##### Discussion.

*Chokwenema* gen. n. resembles the African genus *Batwanema* gen. n. by having a similar arrangement of the cervical spines: first row of eight rectangular scales gradually bifurcating, turning into pointed spines. The genus differs by its genital tract didelphic-amphidelphic *vs.* monodelphic-prodelphic in *Batwanema* gen. n. The procorpus is sub-cylindrical in *Chokwenema* gen. n. in opposition to the clavate procorpus of *Batwanema* gen. n. Moreover, *Chokwenema* gen. n. posses a single, evident truncate first cephalic annule, slightly inflated instead of the two hardly marked annuli of *Batwanema* gen. n., barely expanded.

*Lepidonema* and *Salesia* also bear scale-like projections in the cervical cuticle and shows a didelphic-amphidelphic genital system (Travassos and Kloss 1958). In addition, *Lepidonema* have a sub-cylindrical procorpus. *Chokwenema* gen. n. can be differentiated from both by the arrangement of the cervical spines with eight scales in the first row and the characteristic bifurcation of these, their number increasing towards the end of the spiny region. *Lepidonema* and *Salesia* present more elements in their first rows of spines, that are not bifurcated. Moreover, *Salesia* present a clavate procorpus *vs.* the sub-cylindrical of *Chokwenema* gen. n.

The other digonant genus with spines in the cuticle and sub-cylindrical procorpus is *Soaresnema* Travassos & Kloss, 1958, which can be segregated from *Chokwenema* gen. n. by lacking scales in the cervical cuticle, by the spines forming transverse rows *vs.* opposite rows and by the larger number of elements in the first row (16) *vs.* eight in *Chokwenema* gen. n.

##### Type host.

*Didimoides* cf. *parastictus* (Imhoff, 1843) (Coleoptera: Passalidae).

##### Other host.

*Pentalobus barbatus* (Fabricius, 1801) (Coleoptera: Passalidae).

##### Site.

gut caeca.

##### Type locality.

Mongwalu, Ituri province, Democratic Republic of Congo.

##### Etymology.

Specific epithet derived from the Greek *lepidos*: scale and *phoreus*: to bear, after the scale-like projections of the cervical cuticle.

## Supplementary Material

XML Treatment for Batwanema

XML Treatment for Batwanema congo

XML Treatment for Chokwenema

XML Treatment for Chokwenema lepidophorum
